# Supplementary material for: Human Health during Space Travel: State-of-the-Art Review
Source: Cells. 2022 Dec 22;12(1):40. doi: 10.3390/cells12010040 (PMC9818606; doi:10.3390/cells12010040)
Supplement: Supplementary file 1 [file cells-12-00040-s001.zip › cells-2040859-supplementary.pdf]

Supplementary Table S1: Summary of gut microbial alteration during spaceflight

Microbiome	species	Associated conditions	Changes
Gut	Akkermansia[1, 2]	Increase in the inflammatory immune response	↓
Gut	*Fusicatenibacter*[1]	Increase in the inflammatory immune response	↓
Gut	*Pseudobutyvibrio*[1]	Increase in the inflammatory immune response	↓
Gut	*Parasutterella*[2]	Chronic intestinal inflammation in patients with inflammatory bowel disease (IBD)	↑
Gut	*Bifidobacteria* [3-5]	Immune system injury	↓
Gut	*Lactobacilli*[3]	Immune system injury	↓
Gut	*Bacteroides*[3]	Opportunistic pathogen	↑
Gut	*Salmonella*[6]	Increased virulence and antibiotic resistance (in-vitro)	
Gut	*Escherichia coli*[6]	Increased virulence and antibiotic resistance (in-vitro)	
Gut	*Clostridiaceae*[7]	Increase in the inflammatory immune response	↓
Gut	Firmicutes to Bacteroidetes (F/B) ratio[8]	Ratio changes noted in response to spaceflight, however ratio returned to preflight levels within weeks of landing	↑
Gut	*Faecalibacterium prausnitzii,*[2]	Decreased production of some short-chain fatty acids (SCFA)	↓
Gut	*Parasutterella*[1]	Chronic intestinal inflammation in patients with inflammatory bowel disease (IBD)	↑
Skin	Proteobacteria (mostly Gamma and Betaproteobacteria)[1]	Inversely associated with inflammation and allergy sensitization	↓
Skin	Firmicutes[1]		↑
Skin	*Staphylococcus aureus*[1, 9-11]	Spaceflight-isolated *staphylococcus* species were resistant to at least 1 antibiotic	↑
Skin	*Streptococcal*		↑
Skin	*Pseudomonas*[9, 10]		↑
Skin	Enterobacteriaceae[9]		↑
Nasopharynx	*Staphylococcus*[1, 12]	Prevalence of *staphylococcus aureus* may be associated with chronic rhinosinusitis, allergic rhinitis, nasal polyps, and asthma	↑
Nasopharynx	*Corynebacterium*		↑
Nasopharynx	*Bifidobacterium*[1, 13]	May have a protective effect on allergic rhinitis	↑

Supplementary Table S2: Summary of immune/cytokine changes during spaceflight

Immune/cytokines	Inflight (long duration) (at least 6 months)	Post spaceflight (after returning to Earth)
CXCL8[1]	↑	
IL-8[1, 14]	↑	
IL-1b[1]	↑	
IL-1ra[1, 14]	↑	
TNFa[1, 15]	↑	
IL-6[15, 16]		↑
IL-3[17]	↑	
IL-7[17]	↑	
IL-15[17]	↑	
IL-12p40[17]	↑	
TGF-ß1 and TGF-ß2[17]	↑	
IL-10[15, 16]		↑
IL-1ra[15, 16]		↑
CCL2[15, 16]		↑
CRP[15, 16]		↑
Saliva GM-CSF[17]		↓
Saliva IL-12p70[17]		↓
Saliva IL-10 [17]		↓
Saliva IL-13[17]		↓

Supplementary Table S3: Summary of diet recommendation during spaceflight

**Diet and Supplements**	
Protein intake, % of kcal	12–15% of total daily energy intake
Carbohydrate intake, % of kcal	50% to 55% of total daily energy intake
Fat intake, % of kcal	30–35% of total daily energy intake
Water	> 2 liters per day
Total dietary fiber, g/d	10–25 grams per day
Fruits and vegetables	Recommended

Vitamin D supplements	Recommended

Supplements of A, B6, B12, C, E, K, Biotin, Folic acid are not recommended at this time due to insufficient evidence[18, 19]

Supplementary Table S4: Summary of AI technology and potential applications in space

Problem	Technology	Potential applications
Imaging	The Space-Feasibility Body Composition and Body Shape Analysis for Long Duration Missions (ASTRO3DO) project[20]	3D optical scanners using AI to monitor an astronaut's total body compositions (lean, fat, percent fat, BMD), regional compositions (visceral fat, subcutaneous fat, lumbar spine BMD)
Imaging	AMO project[20, 21]<br><br>AMO MDSS computer system	Virtual medical officer (Tietronix Intelligent Medical Crew Assistant), Augmented Reality enhanced ultrasound improves the reliability of ultrasound image interpretations
Imaging	AI-enhanced ultrasound[22]	AI-enhanced clinical-grade images for inflight echocardiography (UltraSight with GE Healthcare) and point of care ultrasound (GE Healthcare Vsan Air wireless transducer)
Imaging	Deep learning enhanced microtomography[23]	CMCT system has great promise for temporal bone imaging
Monitoring	Random forest<br>Bayes network algorithm[24]	AI could monitor or predict colorectal cancer based on analysis of fecal and gut microbiota
Monitoring	MitoMo (Proprietary algorithm)[25]	AI could monitor microscopic changes in mitochondria
Monitoring	DeepMAge (Deep learning)[26]	AI could monitor microscopic changes in epigenetics
Monitoring	XGBoost[27-29]	AI could monitor small changes in telomere-length dynamics
Monitoring and prediction	Pro-inflammatory cell phenotypes and plasma cytokines[30, 31]	AI could monitor microscopic changes in levels of inflammatory cytokines
Monitoring and prediction	Proprietary ML algorithm[32, 33]	AI could monitor intracranial pressure levels using ultrasonography of the optic nerve sheath diameter in order to predict SANS development
Biosensors	Generative AI[34]<br><br>Wearable technology[35]	Synthetic Astroskin Data (detect electrocardiographic changes)
Prediction	CRISP 2.0 model<br><br>Intel's Open Federated Learning (OpenFL) framework[36, 37]	AI could predict the genes or novel biomarkers for the development of cancer secondary to radiation exposure based on data from a

		combination of mouse, human, and genetic studies
Unknown or novel risk factors prediction	CRISP[37, 38]<br><br>Gradient boosting decision trees[39]<br><br>Logistic regression[40]	AI could identify 'hidden' risk factors from simulated microgravity and radiation exposure
Prediction	Proprietary ML algorithm[41]	AI could detect the risk of cerebrovascular disease using estimates of carotid-femoral pulse wave velocity values obtained from doppler ultrasound
Prediction	Hybrid method (deep learning and empirical mode decomposition)[42]	AI could potentially predict radiation dose estimations
Prediction	Support Vector Machines[43]	AI potentially predict genomic signatures of human radiation response
Device, organ	AI-enhanced 3D printing[44, 45]	In a microgravity environment,<br>3D-printed soft tissues will maintain their shape.
Medical Decision Support System	Autonomous Medical Operations (AMO)[20]	AI-assistance to aid astronauts in the timely diagnosis (e.g., ultrasound) and treatment of emergent conditions (e.g., advisory software, virtual assistance)
Triage	Operations (AMO)[20]	AI assistant that assigns degrees of urgency to a medical scenario
Biosensors	Wearable devices, motion sensors[35]	A combination of AI and wearable technology could monitor vital signs, sleep cycle
Interpretation	K-Means clusters and Support Vector Machine[46]	AI could potentially provide a complementary approach to classic statistical analysis and enable a more robust assessment of microstructures (e.g., bone microarchitecture) using microCT

REFERENCES

1. Voorhies AA, Mark Ott C, Mehta S, Pierson DL, Crucian BE, Feiveson A, Oubre CM, Torralba M, Moncera K, Zhang Y, Zurek E, Lorenzi HA. Study of the impact of long-duration space missions at the international space station on the astronaut microbiome. *Scientific Reports*. 2019;9:9911
2. Turroni S, Magnani M, KC P, Lesnik P, Vidal H, Heer M. Gut microbiome and space travelers' health: State of the art and possible pro/prebiotic strategies for long-term space missions. *Frontiers in Physiology*. 2020;11

3. Liu Z, Luo G, Du R, Sun W, Li J, Lan H, Chen P, Yuan X, Cao D, Li Y, Liu C, Liang S, Jin X, Yang R, Bi Y, Han Y, Cao P, Zhao W, Ling S, Li Y. Effects of spaceflight on the composition and function of the human gut microbiota. *Gut Microbes*. 2020;11:807-819
4. Gibson GR, Wang X. Regulatory effects of bifidobacteria on the growth of other colonic bacteria. *The Journal of applied bacteriology*. 1994;77:412-420
5. Liévin V, Peiffer I, Hudault S, Rochat F, Brassart D, Neeser JR, Servin AL. Bifidobacterium strains from resident infant human gastrointestinal microflora exert antimicrobial activity. *Gut*. 2000;47:646-652
6. Saei AA, Barzegari A. The microbiome: The forgotten organ of the astronaut's body--probiotics beyond terrestrial limits. *Future microbiology*. 2012;7:1037-1046
7. Casero D, Gill K, Sridharan V, Koturbash I, Nelson G, Hauer-Jensen M, Boerma M, Braun J, Cheema AK. Space-type radiation induces multimodal responses in the mouse gut microbiome and metabolome. *Microbiome*. 2017;5:105
8. Garrett-Bakelman FE, Darshi M, Green SJ, Gur RC, Lin L, Macias BR, McKenna MJ, Meydan C, Mishra T, Nasrini J, Piening BD, Rizzardi LF, Sharma K, Siamwala JH, Taylor L, Vitaterna MH, Afkarian M, Afshinnekoo E, Ahadi S, Ambati A, Arya M, Bezdan D, Callahan CM, Chen S, Choi AMK, Chlipala GE, Contrepois K, Covington M, Crucian BE, De Vivo I, Dinges DF, Ebert DJ, Feinberg JI, Gandara JA, George KA, Goutsias J, Grills GS, Hargens AR, Heer M, Hillary RP, Hoofnagle AN, Hook VYH, Jenkinson G, Jiang P, Keshavarzian A, Laurie SS, Lee-McMullen B, Lumpkins SB, MacKay M, Maienschein-Cline MG, Melnick AM, Moore TM, Nakahira K, Patel HH, Pietrzyk R, Rao V, Saito R, Salins DN, Schilling JM, Sears DD, Sheridan CK, Stenger MB, Tryggvadottir R, Urban AE, Vaisar T, Van Espen B, Zhang J, Ziegler MG, Zwart SR, Charles JB, Kundrot CE, Scott GBI, Bailey SM, Basner M, Feinberg AP, Lee SMC, Mason CE, Mignot E, Rana BK, Smith SM, Snyder MP, Turek FW. The nasa twins study: A multidimensional analysis of a year-long human spaceflight. *Science (New York, N.Y.)*. 2019;364
9. Taylor GR. Recovery of medically important microorganisms from apollo astronauts. *Aerospace medicine*. 1974;45:824-828
10. Ilyin VK. Microbiological status of cosmonauts during orbital spaceflights on salyut and mir orbital stations. *Acta astronautica*. 2005;56:839-850
11. Reidt U, Helwig A, Plobner L, Lugmayr V, UlrichTreutlein, Kharin S, Smirnov Y, Novikova N, Lenic J, Fetter V, Hummel T. Study of initial colonization by environmental microorganisms in the russian segment of the international space station (iss). *Gravitational and space biology bulletin: publication of the American Society for Gravitational and Space Biology*. 2014;2:46-57
12. Ramakrishnan VR, Feazel LM, Abrass LJ, Frank DN. Prevalence and abundance of staphylococcus aureus in the middle meatus of patients with chronic rhinosinusitis, nasal polyps, and asthma. *International forum of allergy & rhinology*. 2013;3:267-271
13. Singh A, Hacini-Rachinel F, Gosoniu ML, Bourdeau T, Holvoet S, Doucet-Ladeveze R, Beaumont M, Mercenier A, Nutten S. Immune-modulatory effect of probiotic bifidobacterium lactis ncc2818 in individuals suffering from seasonal allergic rhinitis to grass pollen: An exploratory, randomized, placebo-controlled clinical trial. *European journal of clinical nutrition*. 2013;67:161-167
14. Crucian BE, Makedonas G, Sams CF, Pierson DL, Simpson R, Stowe RP, Smith SM, Zwart SR, Krieger SS, Rooney B, Douglas G, Downs M, Nelman-Gonzalez M, Williams

TJ, Mehta S. Countermeasures-based improvements in stress, immune system dysregulation and latent herpesvirus reactivation onboard the international space station - relevance for deep space missions and terrestrial medicine. *Neuroscience and biobehavioral reviews*. 2020;115:68-76
15. Crucian BE, Zwart SR, Mehta S, Uchakin P, Quiriarte HD, Pierson D, Sams CF, Smith SM. Plasma cytokine concentrations indicate that in vivo hormonal regulation of immunity is altered during long-duration spaceflight. *Journal of interferon & cytokine research : the official journal of the International Society for Interferon and Cytokine Research*. 2014;34:778-786
16. Gertz ML, Chin CR, Tomoiaga D, MacKay M, Chang C, Butler D, Afshinnekoo E, Bezdan D, Schmidt MA, Mozsary C, Melnick A, Garrett-Bakelman F, Crucian B, Lee SMC, Zwart SR, Smith SM, Meydan C, Mason CE. Multi-omic, single-cell, and biochemical profiles of astronauts guide pharmacological strategies for returning to gravity. *Cell reports*. 2020;33:108429
17. Krieger SS, Zwart SR, Mehta S, Wu H, Simpson RJ, Smith SM, Crucian B. Alterations in saliva and plasma cytokine concentrations during long-duration spaceflight. *Frontiers in Immunology*. 2021;12
18. Nutritional NAaSAJSC, requirements for International Space Station (ISS) missions up to 360 days. Houston TNAaSALBJSC, JSC-28038 RN.
19. Nutrition NAaSAJSC, Requirements S, and Operating Bands for Exploration Missions. JSC, Document #63555. Houston TN.
20. NASA. Space Technology Game Changing Development Autonomous Medical Operations. Available from: https://www.nasa.gov/sites/default/files/atoms/files/amo_fs_180502_0.pdf.
21. NASA. Autonomous Medical Operations Intelligent Medical Crew Assistant (IMCA). Available from: https://sbir.nasa.gov/SBIR/abstracts/20/sbir/phase1/SBIR-20-1-H12.05-6577.html.
22. Cardiovascular Ultrasound. AI Enabled Wireless Cardiac Point-of-Care-Ultrasound Will be Used by Astronauts in Space. Available from: https://www.dicardiology.com/content/ai-enabled-wireless-cardiac-point-care-ultrasound-will-be-used-astronauts-space.
23. Li M, Fang Z, Cong W, Niu C, Wu W, Uher J, Bennett J, Rubinstein JT, Wang GE. Clinical micro-ct empowered by interior tomography, robotic scanning, and deep learning. *IEEE access : practical innovations, open solutions*. 2020;8:229018-229032
24. Ai L, Tian H, Chen Z, Chen H, Xu J, Fang JY. Systematic evaluation of supervised classifiers for fecal microbiota-based prediction of colorectal cancer. *Oncotarget*. 2017;8:9546-9556
25. Zahedi A, On V, Phandthong R, Chaili A, Remark G, Bhanu B, Talbot P. Deep analysis of mitochondria and cell health using machine learning. *Scientific Reports*. 2018;8:16354
26. Galkin F, Mamoshina P, Kochetov K, Sidorenko D, Zhavoronkov A. Deepmage: A methylation aging clock developed with deep learning. *Aging Dis*. 2021;12:1252-1262
27. Luxton JJ, McKenna MJ, Lewis A, Taylor LE, George KA, Dixit SM, Moniz M, Benegas W, Mackay MJ, Mozsary C, Butler D, Bezdan D, Meydan C, Crucian BE, Zwart SR, Smith SM, Mason CE, Bailey SM. Telomere length dynamics and DNA damage responses associated with long-duration spaceflight. *Cell reports*. 2020;33:108457

28. Luxton JJ, McKenna MJ, Taylor LE, George KA, Zwart SR, Crucian BE, Drel VR, Garrett-Bakelman FE, Mackay MJ, Butler D, Foox J, Grigorev K, Bezdan D, Meydan C, Smith SM, Sharma K, Mason CE, Bailey SM. Temporal telomere and DNA damage responses in the space radiation environment. *Cell reports*. 2020;33:108435
29. Luxton JJ, McKenna MJ, Lewis AM, Taylor LE, Jhavar SG, Swanson GP, Bailey SM. Telomere length dynamics and chromosomal instability for predicting individual radiosensitivity and risk via machine learning. *Journal of personalized medicine*. 2021;11
30. Rostam HM, Reynolds PM, Alexander MR, Gadegaard N, Ghaemmaghami AM. Image based machine learning for identification of macrophage subsets. *Sci Rep*. 2017;7:3521
31. Patterson BK, Guevara-Coto J, Yogendra R, Francisco EB, Long E, Pise A, Rodrigues H, Parikh P, Mora J, Mora-Rodríguez RA. Immune-based prediction of covid-19 severity and chronicity decoded using machine learning. *Front Immunol*. 2021;12:700782
32. Miyagawa T, Sasaki M, Yamaura A. Intracranial pressure based decision making: Prediction of suspected increased intracranial pressure with machine learning. *PloS one*. 2020;15:e0240845
33. Lochner P, Czosnyka M, Naldi A, Lyros E, Pelosi P, Mathur S, Fassbender K, Robba C. Optic nerve sheath diameter: Present and future perspectives for neurologists and critical care physicians. *Neurological sciences : official journal of the Italian Neurological Society and of the Italian Society of Clinical Neurophysiology*. 2019;40:2447-2457
34. https://ntrs.nasa.gov/api/citations/20190000238/downloads/20190000238.pdf.
35. Krittanawong C, Rogers AJ, Johnson KW, Wang Z, Turakhia MP, Halperin JL, Narayan SM. Integration of novel monitoring devices with machine learning technology for scalable cardiovascular management. *Nature reviews. Cardiology*. 2021;18:75-91
36. Intel. Intel AI Mentors Seek to Improve Astronaut Health. Available from: https://www.intel.com/content/www/us/en/newsroom/news/intel-ai-mentors-seek-improve-astronaut-health.html#gs.k24fbq.
37. Budd S, Blaas A, Hoarfrost A, Khezeli K, Silva KD, Soboczenski F, Mackintosh G, Chia N, Kalantari J. Prototyping crisp: A causal relation and inference search platform applied to colorectal cancer data. *2021 IEEE 3rd Global Conference on Life Sciences and Technologies (LifeTech)*. 2021:517-521
38. https://drive.google.com/file/d/1Kyu9IiAp0jeRTW2wYc9YqaP25J7oVwVb/view.
39. Madakkatel I, Zhou A, McDonnell MD, Hyppönen E. Combining machine learning and conventional statistical approaches for risk factor discovery in a large cohort study. *Scientific Reports*. 2021;11:22997
40. Li M, Zhang Z, Cao W, Liu Y, Du B, Chen C, Liu Q, Uddin MN, Jiang S, Chen C, Zhang Y, Wang X. Identifying novel factors associated with covid-19 transmission and fatality using the machine learning approach. *The Science of the total environment*. 2021;764:142810
41. Tavallali P, Razavi M, Pahlevan NM. Artificial intelligence estimation of carotid-femoral pulse wave velocity using carotid waveform. *Sci Rep*. 2018;8:1014
42. Götz TI, Schmidkonz C, Chen S, Al-Baddai S, Kuwert T, Lang EW. A deep learning approach to radiation dose estimation. *Physics in medicine and biology*. 2020;65:035007
43. Zhao JZL, Mucaki EJ, Rogan PK. Predicting ionizing radiation exposure using biochemically-inspired genomic machine learning. *F1000Research*. 2018;7:233
44. Goh GD, Sing SL, Yeong WY. A review on machine learning in 3d printing: Applications, potential, and challenges. *Artificial Intelligence Review*. 2021;54:63-94

45. Success: 3d bioprinter in space prints with human heart cells. Available from: https://techshot.com/success-3d-bioprinter-in-space-prints-with-human-heart-cells/.
46. Coulombe JC, Mullen ZK, Lynch ME, Stodieck LS, Ferguson VL. Application of machine learning classifiers for microcomputed tomography data assessment of mouse bone microarchitecture. *MethodsX*. 2021;8:101497
